# Novel Synthesis of Nano Mg(OH)_2_ by Means of Hydrothermal Method with Different Surfactants

**DOI:** 10.3390/nano13030454

**Published:** 2023-01-22

**Authors:** Zahra Rajabimashhadi, Rahim Naghizadeh, Ashkan Zolriasatein, Carola Esposito Corcione

**Affiliations:** 1 School of Metallurgy and Materials Engineering, Iran University of Science and Technology, Tehran 16846-13114, Iran; 2 Non-Metallic Materials Research Department, Niroo Research Institute, Tehran 1466-5517, Iran; 3 Department of Innovation Engineering, University of Salento, 73100 Lecce, Italy

**Keywords:** hydrothermal, surface modification, morphology, particle size distribution

## Abstract

Magnesium hydroxide (MOH) is a widely used inorganic chemical owing to its various properties. Hence, researchers have long studied its synthesis and its unique features. However, the morphological consequences have rarely been studied. Despite having several benefits for synthesizing nanoparticles, the hydrothermal method’s main drawbacks are its lengthy processing time and the high cost of raw materials. This research aimed to use more easily obtainable raw materials in a reasonably short time to synthesize MOH in various morphologies. For this purpose, we prepared different samples using the same hydrothermal method to investigate the effects of the precursor and surfactant on the structure, morphology, and size of MOH particles. The results of XRD and FTIR analysis demonstrated that a temperature of 180 °C and a duration of 18 h is not sufficient for MgO as a precursor to obtaining MOH in the hydrothermal method. However, in the presence of different surfactants, MgCl${}_{2}$ resulted in nanoparticles with hexagonal structure and plate, flake, spherical, and disc morphologies.

## 1. Introduction

**Figure 1:**
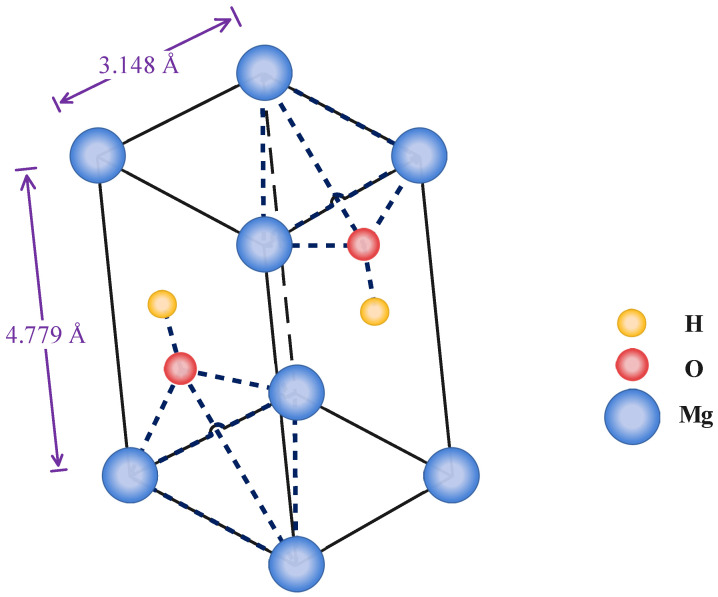
Crystalline lattice of MOH.

Magnesium hydroxide (MOH) is a mineral that has several beneficial and specific features. These characteristics have a broad range of applications in science and, more importantly, in the practical world [1]. As illustrated in Figure 1, MOH crystallizes in tetragonal 3D space with (a) and (c) lattice parameters of 3.148, and 4.779 Å, respectively. Each ${\mathrm{Mg}}^{2+}$ is coordinated by six ${\mathrm{OH}}^{-}$ to create a Mg-OH octahedral that shares 2D faces, leading to a layered structure in the MOH crystalline lattice. However, in a triangular geometry, each ${\mathrm{OH}}^{-}$ anion is surrounded by three ${\mathrm{Mg}}^{2+}$ cations [2,3]. The oxygen atoms in ${\mathrm{OH}}^{-}$ anions are arranged above and below the ${\mathrm{Mg}}^{2+}$ cations planes, with O-H bonds perpendicular to them. Hydrogen bonding finalizes the extended crystal structure in the third dimension, weakly bonding the hexagonal close-packed (HCP) anion layers together, with the shortest H…H interlayer distance of 1.97 Å. The hydroxide hydrogens within one layer faced towards the center of the triangular plane created by three OH bonds in the next layer in the …ABAB… HCP layers [3,4,5]. On the other hand, MOH is a fire-resistant and insulating substance as well as an antibacterial agent. By heating the MOH to temperatures above 350 °C, according to Equation (1), a dehydration reaction occurs, during which MOH is converted to magnesium oxide and water [6,7,8,9,10].

The rationale for the widespread use of MOH as a non-toxic commercial flame retardant is its ability to decompose endotherms, release water and produce magnesium oxide without any toxic by-products. Furthermore, MOH found its place as an acidic waste neutralizer, medicinal excipient, paper conservation, chemical sensor, and the most important precursor for the synthesis of Nano-MOH [11,12].

(1) $$\mathrm{Mg}{\left(\mathrm{OH}\right)}_{2}\to \mathrm{MgO}+H_{2}O\;\Delta H^{\circ }\left(298\;K\right)=98.4\;\mathrm{kJ}\;{\mathrm{mol}}^{-1}H_{2}O$$

There are several methods used to synthesize Nano-MOH, including hydrothermal, solvothermal, sol–gel, chemical precipitation, electrochemical, and microwave techniques. Moreover, surfactants and synthesizing agents have also been used to produce Nano-MOH with various morphologies, including plates to tubes, rods, sheets, and flakes, as well as spherical formations [13,14,15,16,17,18,19,20,21,22]. Product properties, such as structure, morphology, and dispersion, can be considerably influenced by different process parameters. So, many experimental kinds of research are performed to determine the reaction conditions that will have the most desirable impact on the product. The influence of the concentrations, rate, proportions of reagent, the temperature of the precipitation and calcination processes, and the drying technique are the most commonly studied [23,24,25,26]. In the chemical precipitation method, MOH is precipitated from salt solutions using a precipitating agent, which is typically a strong base such as ammonia or sodium hydroxide. Inorganic salts, such as magnesium chloride, magnesium sulfate, and magnesium nitrate are the most widely utilized magnesium precursors in this method [27,28,29,30,31,32,33].

Hydrothermal synthesis is a method for crystallizing materials that involves chemical reactions in aqueous solutions under pressure and at a temperature above boiling point. In this approach, water has two critical roles: first, it dissolves the reagents, and secondly, it transfers the pressure through all the solution’s components. The main advantages of hydrothermally synthesized powders are the low degree of agglomeration and perfect control of crystallite size and morphology [21,34]. The hydrothermal method of producing MOH is performed in a stainless steel autoclave with a thin layer of PTFT on the inside to protect it from the corrosive environment. Mg precursor and precipitating agent are placed in the autoclave, and then solvent is added up to two thirds of the reactor’s total capacity. The autoclave is sealed completely and subjected to high temperatures for an extended time. Finally, the synthesized powder is washed and dried after cooling [35]. Li was the first to publish the solvothermal synthesis of Nano-rod MOH in 2000. He employed ethylene diamine as a templating agent and Mg metal as a magnesium precursor in a process known as “soft templating” (Equation (2)) [36].

(2) $$\mathrm{Mg}+2H_{2}O\to \mathrm{Mg}{\left(\mathrm{OH}\right)}_{2}+H_{2}$$

Ding, also presented a significant study on MOH hydrothermal synthesis parameters in 2001. He described the synthesis of Nano-MOH that allowed for selective customization of morphology and size. By altering the aqueous solvent composition as well as the magnesium source (Mg, MgSO${}_{4}$, and Mg(NO${}_{3}$)${}_{2}$ and modifying the hydrothermal reaction conditions, rod, tube, needle, or lamellar-like nanoparticles were obtained [13]. Considering the wide variety of chemical parameters used in the hydrothermal method, it is difficult to understand their exact role in a single experiment and even more challenging to consider their interactions across multiple. The nucleation and crystal growth are usually initiated by the ability of ligands (molecular additives) to form coordination complexes with ${\mathrm{Mg}}^{2+}$ cations presented from magnesium precursors (metal, salt, or oxidized). The coordination behavior of the different ligands to the center metal, as well as the shape of the precursor, might have an impact on the formation of the rod, needle, tube, or other lamellar morphologies [27,28,29].

MOH particles tend to join into complex microstructures to build aggregates larger than 1 µm. Many properties of polymeric materials, such as mechanical characteristics, are harmed by large agglomerates of irregularly shaped particles, which reduce their resistance to bending and stretching. Indeed, it is critical to choose appropriate conditions and procedures for chemical synthesis in order to achieve desirable morphological and microstructural influences [37]. According to the most recent literature findings, the ultimate characteristics of the product materials—most notably, their shape—are largely reliant on the type of precursor and precipitating agent utilized. In addition to the required reagents, it is becoming more usual for modifying compounds to be used. The in situ method is performed in processes that synthesize nanoparticles from aqueous precursor solutions with the modifying agent added during the precipitation step. In this way, with correct selection of surfactant, desirable changes in morphology and dispersion can be obtained [30,31,32,33].

In this study, the main objective for MOH synthesis was to develop new synthetic approaches that are rapid, simple, energy efficient, and allow for fine particle size and morphology control. In this regard, MOH was synthesized from two precursors of magnesium oxide and magnesium chloride by hydrothermal method (180 °C-18 h). Additionally, four surfactants, including CTAB, PEG500, Gelatin, and Oleic Acid, were used to achieve different morphologies and comparison of these materials’ effects on morphology and particle size of the MOH product. We selected inexpensive, available, non-toxic, and active surfactants to obtain Nano-MOH via a simple and easy method. In addition, some of these surfactants were investigated in previous research studies to modify other nanoparticles (not MOH). On the other hand, the active agents in these four modifiers were different (see Table 1) from each other. Therefore, based on this study, we expected to see different results in the final product. The novelty of this research is that it investigates the possibility of Nano-MOH synthesis via MgO and MgCl${}_{2}$ in a hydrothermal method. There are a lot of studies focusing on MOH, but to the best of our knowledge, among the literature, no papers have focused on the effect of different surfactants besides pre-sources on morphology and particle size with a mechanism description. Nano powders are examined using XRD, SEM, EDS, and DLS.

## 2. Materials and Methods

The distinguishing properties of MOH make it a special material in many fields such as retardant or reinforcement filler. Different synthesis methods were investigated to obtain Nano-MOH with high purity, low expenses, easy and fast procedure. The shape of this nanoparticle has not been studied compared to other physical and mechanical properties, though it has a great ability to be produced in different morphologies on the Nano-scale. To examine the above-mentioned function, we used more obtainable raw materials in a reasonably short hydrothermal method to synthesize MOH in this study. The major contribution of the proposed research is using different precursors and surfactants in the same hydrothermal method to evaluate the structure, morphology, and size of MOH particles. In this regard, microstructure spectroscopy, phase, and particle characterization were performed. This section presents the materials and methods, including the raw material, synthesis method, sample preparation and analysis tests, in detail.

### 2.1. Materials

Here, two precursors of magnesium chloride (Dr. Mojallali; 7791-18-6; Purity: 98%) and magnesium oxide (SIGMA; 1308-48-4; Purity: 97%) were used to synthesize MOH. Sodium hydroxide (Dr. Mojallali; 1310-73-2; Purity: 95%) was also used as the precipitating agent. Distilled water (Ghatran Shimi; 7732-18-5; Purity: 99.99%) was used as a synthesis solvent, ethanol (Dr. Mojallali; 64-17-5; Purity: 96%) for washing and centrifugation, and 2-Propanol (Dr. Mojallali; 67-63-0; Purity: Extra pure, >99.9%) as a dispersion solvent. The surfactants used to obtain different morphologies are listed in Table 1 [38,39,40].

### 2.2. Sample Preparation

The synthesis of MOH began by producing 1 Molar magnesium precursor solution and 2 Molar sodium hydroxide solution in distilled water. Equations (3) and (4) were used to carry out the synthesis:

(3) $$\mathrm{MgO}+2\mathrm{NaOH}\to \mathrm{Mg}{\left(\mathrm{OH}\right)}_{2}+{\mathrm{Na}}_{2}O$$

(4) $${\mathrm{MgCl}}_{2}\cdot 6H_{2}O+2\mathrm{NaOH}\to \mathrm{Mg}{\left(\mathrm{OH}\right)}_{2}+2\mathrm{NaCl}+6H_{2}O$$

According to Equation (5), one mole of magnesium oxide and two moles of sodium hydroxide react to form one mole of magnesium hydroxide and one mole of sodium oxide. This is a double displacement-type reaction (metathesis) and it might also be an oxidation-reduction reaction.

(5) $$\left[{\mathrm{Mg}}^{2+}\cdot O^{2-}\right]+2\left[{\mathrm{Na}}^{+}\cdot {\mathrm{OH}}^{-}\right]\to \left[{\mathrm{Mg}}^{2+}\cdot 2\left({\mathrm{OH}}^{-}\right)\right]+\left[{\mathrm{Na}}^{+}\cdot 2\left(O^{2-}\right)\right]$$

**Figure 2:**
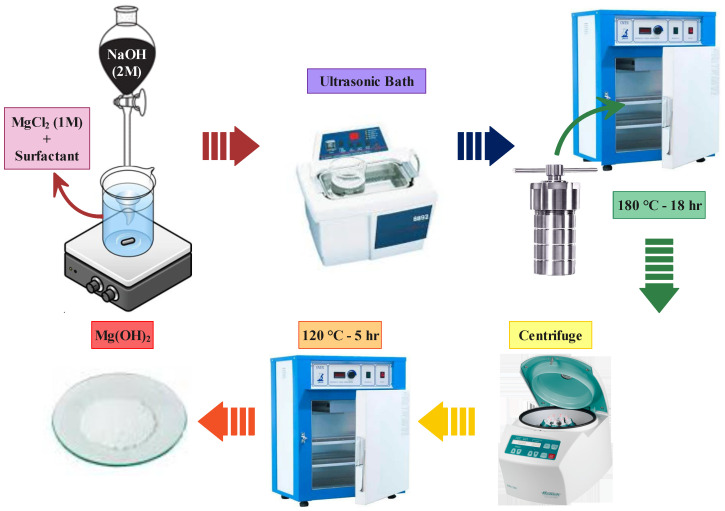
Scheme of MOH powder synthesis by hydrothermal method.

Moreover, 1 %wt. of surfactant was added to magnesium precursor solution, and magnetic stirring was used to homogenize it for a total of 10 min. This solution was then added to magnesium precursor and surfactant solution on a magnetic stirrer using a glass pipette at a controlled rate of 2 mL/min and mixed for 10 min. Next, for 30 min, the final solution was immersed in an ultrasonic bath (power: 400 W) to ensure that the particles were homogeneously dispersed in the solution. This solution was then transferred to a glass vial and placed in a steel autoclave with a Teflon inner chamber. Finally, MOH was synthesized using a hydrothermal method at 180 °C for 18 h. In this method, we used a stainless steel autoclave (hydrothermal reactor with PTFE 250 mL inside container). To eliminate any by-products or unreacted products, the finishing solution was centrifuged at 5000 rpm for 4 min on each run, with distilled water and ethanol (3 runs each). Subsequently, the washed solution was heated in an oven at 120 °C for 5 h to get the synthesized MOH powder, which was then prepared for characterization analyses. Table 2 summarizes the powders’ synthesized data and the synthesis schematic shown in Figure 2.

### 2.3. Tests and Analyses Performed

#### 2.3.1. X-ray Diffractometers (XRD)

XRD was performed to characterize the structure of MOH powder and assess the accuracy of the synthesis. This test was performed with a BOUREVESTNIK DRON-8 X-ray, Petersburg, Russia diffraction device equipped with CuKα monofilament with a wavelength of 0.154 nm, an accelerator voltage of 40 kV, and a current of 30 mA and in the range of 10° to 80° angles with 0.5° steps, a stop time of 1 s per step at 25 °C, and one atmospheric pressure with a copper anode.

#### 2.3.2. Fourier-Transform Infrared Spectroscopy (FTIR)

FTIR Spectroscopy was used to evaluate the chemical bonds of the synthesized Nano-MOH with a FT/IR-6000 FTIR spectrometer, Cremella (LC)-Italy. Indeed, the FTIR spectroscopy of the two groups’ samples was compared together to investigate the effect of different precursors and surfactants in the final product.

#### 2.3.3. Field Emission Scanning Electron Microscopy Analysis (FESEM)

FESEM analysis was used to study the determination of size, morphology, and dispersion of nanoparticles. FESEM analysis of the blends was undertaken using SEM (MIRA3TESCAN-XMU, Kohoutovice, Czech Republic) in high vacuum mode. The radiation source was a Uvc (w5) pencil lamp made by Hach Company, Loveland, CO, USA. The resolution of this device is 1 nm, and its magnification power can reach up to 1 million with the application of 30 volts. All images were captured at 5000× magnification. This was accomplished by grinding MOH powder and dissolving a portion of it in 5 mL 2-propanol. The solution was placed in an ultrasonic bath for 5 min to ensure perfect dispersion of nanoparticles in the solvent and no agglomeration. After that, a drop of solution was scattered on a glass slide and allowed to dry at room temperature. The gold was coated using the Nano-structure COATING DSR model because of the powder’s insulation. Finally, the elemental composition of the samples was evaluated using the EDS test.

#### 2.3.4. Dynamic Light Scattering (DLS)

DLS analysis was performed to investigate the particle size distribution of the synthesized powders using a non-invasive quantitative technique with the Zetasizer Advance Pro device made in the United Kingdom.

## 3. Results and Discussion

### 3.1. XRD

**Figure 3:**
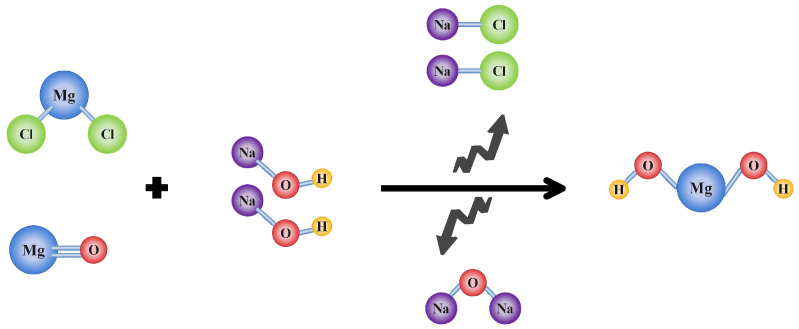
The mechanism of MOH formation from two precursors of MgO and MgCl${}_{2}$.

The reaction of magnesium oxide and magnesium chloride with sodium hydroxide to produce MOH is shown in Figure 3. In order to examine the crystalline phase of the produced Nano-MOH, XRD was used, which included quantities of crystalline structure and crystal size determination (*D*) using the Debay–Scherr method (Equation (6)). *K* is the shape factor (0.9), λ is the X-ray wavelength corresponding to CuKα radiation (0.15406 nm), β is the peak width at half the maximum height, and θ is the Bragg angle [1,2].

(6) D=Kλ/βcosθ

**Figure 4:**
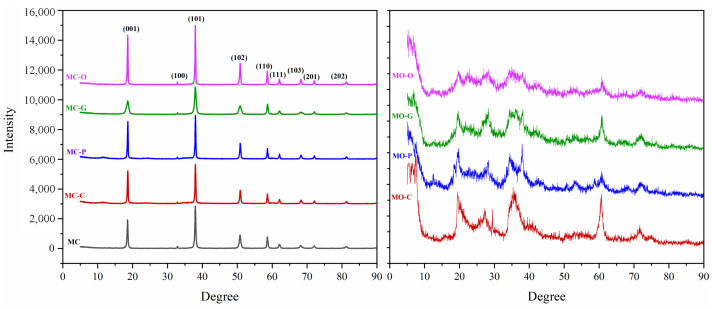
XRD results of powders synthesized from two precursors MgCl${}_{2}$ (**left**) and MgO (**right**).

According to the XRD evaluation of MO-powder samples shown in Figure 4, some diffraction occurred at 18.45°, 37.67°, and 58.78°, corresponding to MOH, and it can be concluded that a small part of magnesium oxide in the reaction participated and became an MOH under these temperature and time conditions. Therefore, it was necessary to carry out the hydrothermal process for longer or at a higher temperature in order to generate a magnesium oxide and sodium hydroxide reaction. Although XRD for MO powders indicated that MOH synthesis was not successful for the MgO precursor, all diffraction peaks of MC-powders were displayed as hexagonal MOH in accordance with the JCPDS 7-0239 standard card with lattice constants of a = 3.144 Å and c = 4.777 (Figure 3). At diffraction angles of 18.45°, 33.24°, 37.67°, 51.12°, 58.78°, 62.34°, 67.86°, 72.15°, and 82.22°, the crystal planes (001), (100), (101), (102), (110), (111), (103), (201), and (201) are observed. The presence of sharp peaks in the XRD pattern, as well as the absence of any impurity peaks, confirms the crystallization of a high-purity single phase of MOH. According to Equation (6), the size of MC, MC-C, MC-P, MC-G, and MC-O at (101) direction were 72.8 ± 4.5 nm, 54.9 ± 5.6 nm, 52.8 ± 5.8 nm, 27.3 ± 2.5 nm and 84.1 ± 6.8 nm, respectively. The orientation of MOH particles is indicated by the intensity of diffraction in planes (001) and (110). The preferred orientation of hexagonal MOH is typically in the direction of the plane (001), and this orientation increases as the intensity of the plates (001) and (110) rises. Additionally, according to the standard card, the (101) intensity should be greater than (001). Otherwise, page (001) is the preferred orientation [19,41].

### 3.2. FTIR

**Figure 5:**
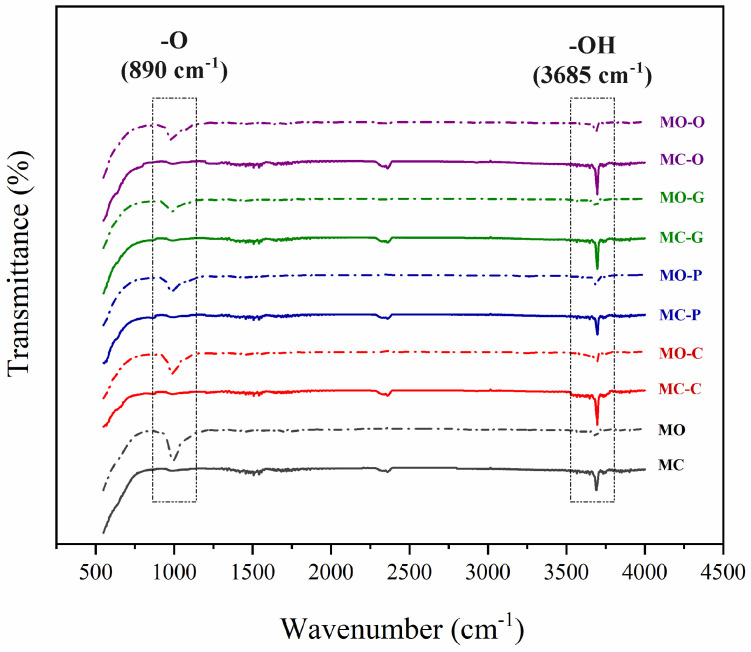
Comparison of FTIR spectroscopy of samples MO and MC.

Figure 5 shows the FTIR spectra of MO and MC samples. The absorption band near 890 cm${}^{-1}$ is characteristic of cubic magnesium oxide, which is attributed to the stretching of –O bond. This peak is only observed in powders synthesized using a magnesium oxide precursor. This spectrum also contains a sharp and intense peak at 3685 cm${}^{-1}$ that is due to the –OH group in both MOH-powders [12,41]. However, its intensity in MC samples is higher than MO. Indeed, a low percent of magnesium oxide contributes to the reaction and turns to MOH under these hydrothermal conditions. Therefore, the –O bond does not remove in MO-powders.

### 3.3. FESEM

**Figure 6:**
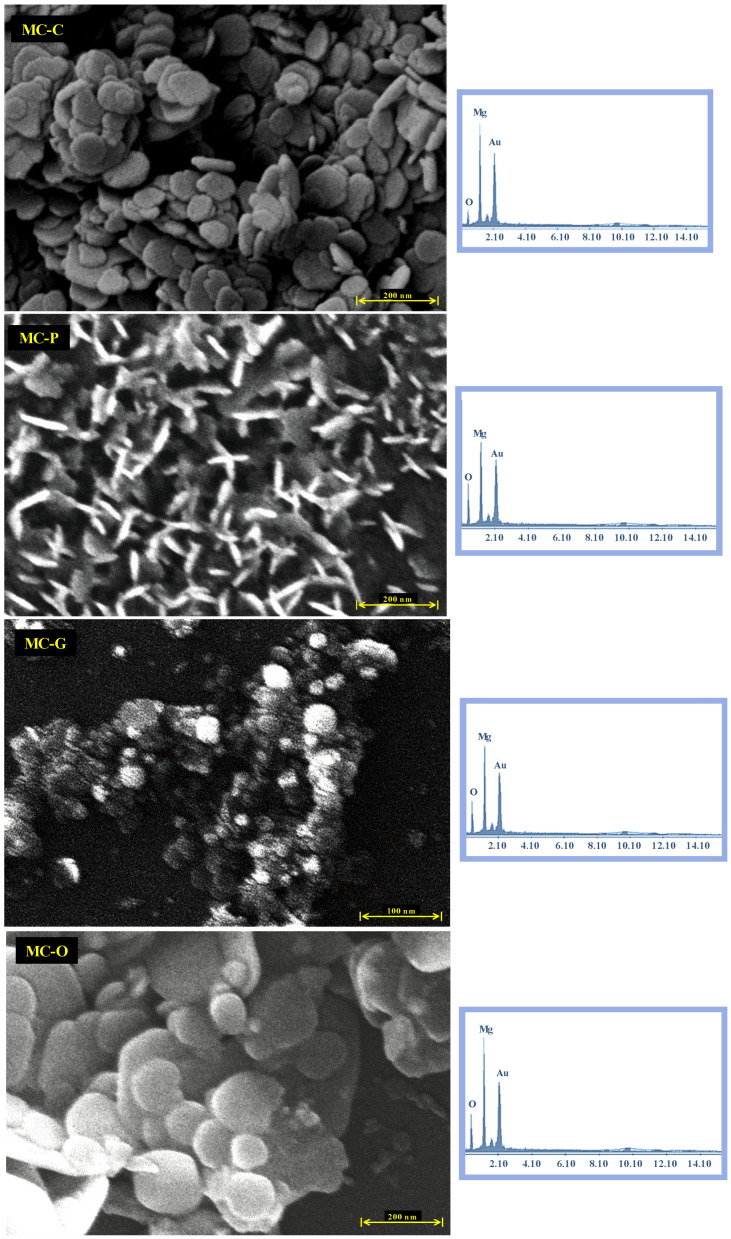
FESEM and EDS analysis of Nano-MOH with various surfactants synthesized from MgCl${}_{2}$.

FESEM images (50 kX) were taken to show the morphology and particle size distribution of Nano-MOH and EDS analysis was performed to investigate the elemental composition of the samples. The initial morphology of the MOH nanoparticles synthesized from magnesium chloride is shown in Figure 6. Although the precursor solutions were added dropwise at a suitable rate of 5 mL/min to synthesize MOH pure powder with a mean size below 100 nm, the lack of surfactant caused aggregation and particle clumping. Peaks in X-ray spectroscopy acquired from powder samples in the bulk state that are related to Mg and O elements without any impurity peaks, excluding Au, which is the coating applied to the surface, demonstrated their excellent purity [14,42].

**Figure 7:**
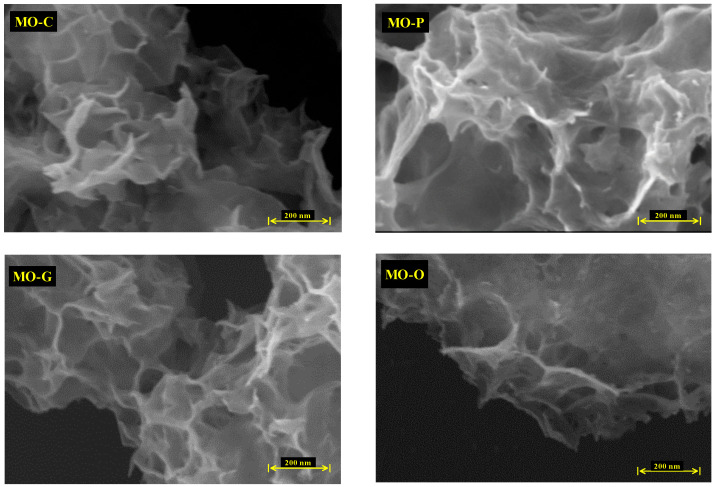
FESEM images of Nano-MOH with various surfactants synthesized from MgO.

The synthesis of Nano-MOH is based on a continuous chemical reaction that results in the formation of primary MOH nucleus, followed by the consumption of magnesium ions in solution and the Ostwald Ripening phenomena, in which larger particles grow as smaller particles are consumed. Next, four compositions as surfactants are utilized with 1 %wt. to study their influence on the morphology and size of the nanoparticles. Meanwhile, surfactants such CTAB, PEG500, Gelatin, and oleic acid contribute with directional growth, and MOH particles come in the shapes of Nano-flake, Nano-plate (thickness less than flake), very fine Nano-spherical form, and Nano-disc (with rounded corners). The extraordinarily small size of the MOH-G sample is observable in the FESEM image, and is explained by the gelatin chain, which is long compared to other surfactants. Considering the presence of gelatin in the solution during synthesis, the first crystals cannot not be overgrown, resulting in the morphology of MOH, which come in the form of small spheres. Another important point is the vertical orientation of MOH-P Nano-plates, which is expected to have a significant impact on the surface properties of nanocomposites. MOH-C is randomly distributed and MOH-O is distributed horizontally. On the other hand, according to the XRD analysis of MO powders in Figure 4, and the FTIR spectrum in Figure 5, it was found that the use of magnesium oxide for the synthesis of MOH by hydrothermal method at 180 °C for 18 h was unsuccessful, and this result is presented in the FESEM images (Figure 7) which show an agglomerated microstructure [17,20].

**Figure 8:**
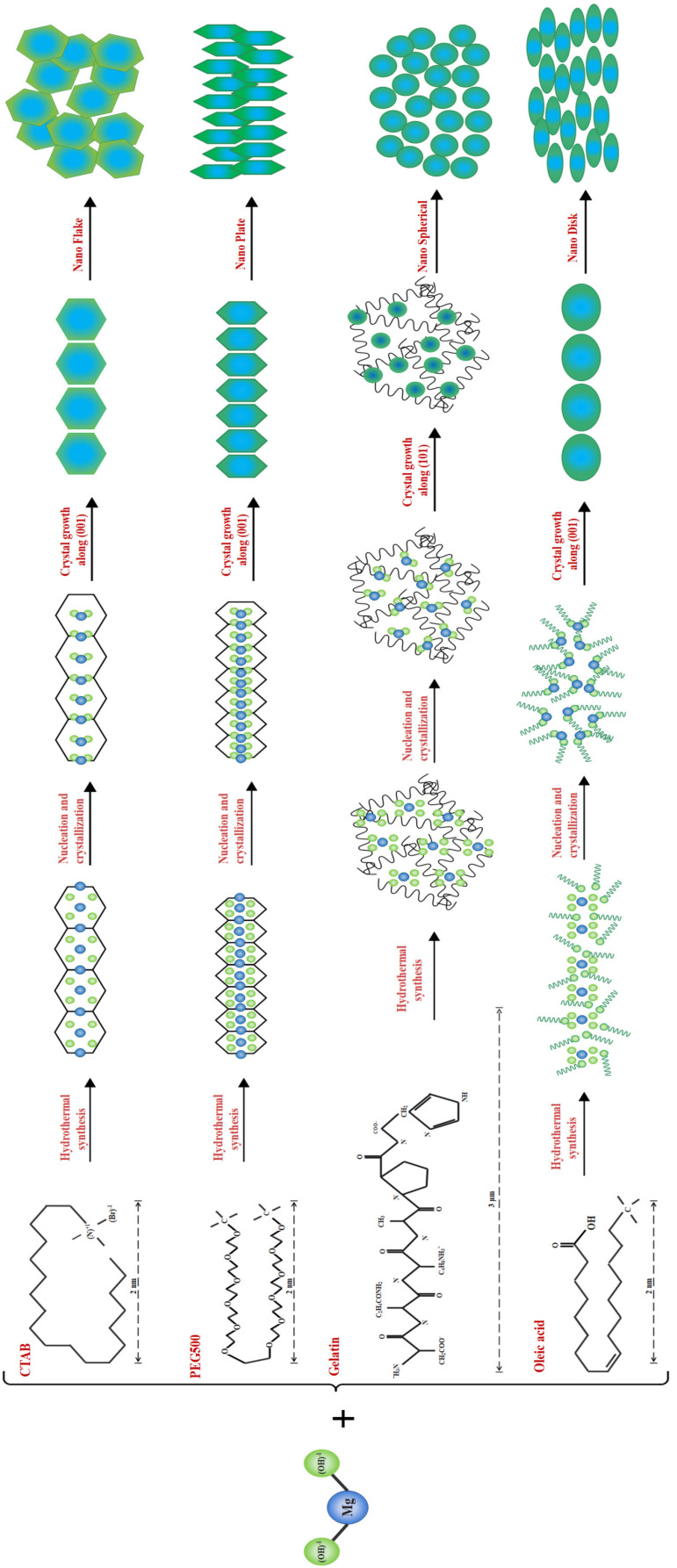
Formation mechanism of four morphologies of Nano-MOH with various surfactants synthesized from MgCl${}_{2}$.

As described in Figure 8, it would be possible to control the morphology of Nano-MOH by using the mentioned surfactants. The presence of CTAB during synthesis allows the formation of MOH-primary nuclei similar to the carbon chain. Subsequently, the growth of the nuclei in the (001) direction leads to the hexagonal MOH plane, which results in the Nano-flake morphology. The same thing happens with the addition of PEG500, but due to the longer carbon chain compared to CTAB, the possibility of primary nuclei growing in the transverse direction is lower, and thinner Nano-plates are created. It is also possible that the presence of a carbon chain with two CH${}_{3}$ agents at both ends and the locking of the nuclei between the chains, the preferential growth of particles occurs along (001) and a certain orientation is observed in the microstructure. In the previous sample, however, the orientation was totally random. The gelatin macromolecule also causes the primary nuclei to grow in all directions during synthesis, preventing overgrowth and thus creating an extra-fine spherical morphology. However, in the case of oleic acid, it can be analyzed that the ${\mathrm{OH}}^{-1}$ factor at the end of the carbon chain ironically exchanges with the ${\mathrm{OH}}^{-1}$ in MOH and the placement of a chain around ${\mathrm{Mg}}^{2+}$ results in rounded edges, creating a Nano-disk [41,42,43].

### 3.4. DLS

**Figure 9:**
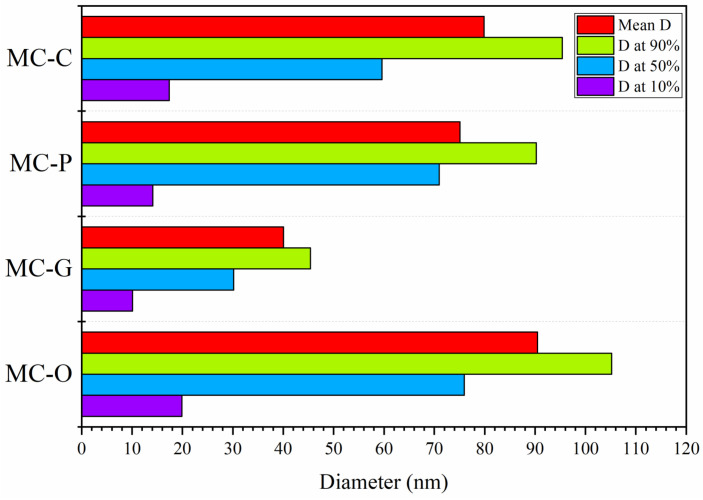
Diameter size variations of Nano-MOH with various surfactants synthesized from MgCl${}_{2}$.

**Figure 10:**
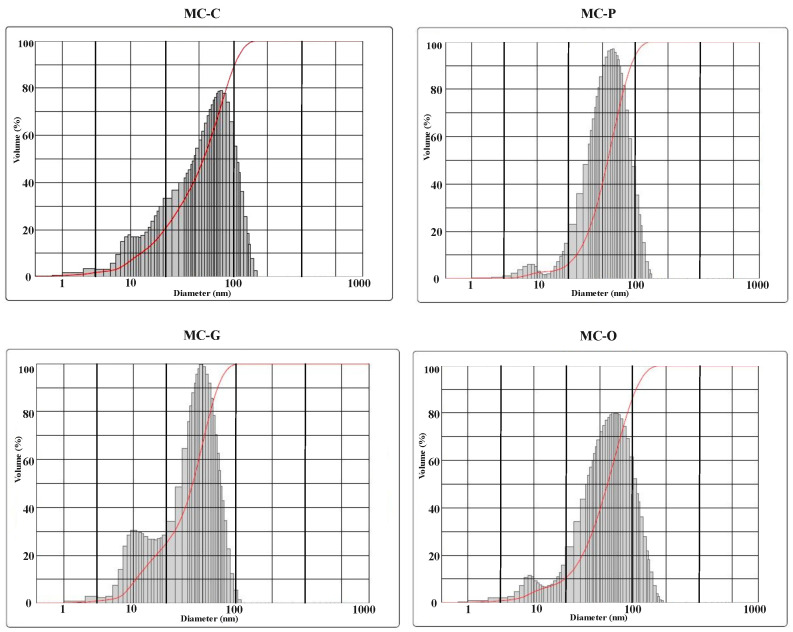
Particle size distribution of Nano-MOH with various surfactants synthesized from MgCl${}_{2}$.

DLS analysis was performed to study the effect of four different surfactants on the particle size of hydrothermally synthesized Nano-MOH using magnesium chloride. The diameters in different volumes of particles and particle size distribution are shown in Figure 9 and Figure 10, respectively. According to Figure 9, DLS analysis of all samples shows a two-peak diagram providing very fine nanoparticles (10–30 nm) and medium (40–100 nm). Using CTAB in the MC-C sample, a uniform and relatively wide curve was obtained, which indicates a wide distribution of particle sizes between 60 and 100 nm. However, by adding PEG as a surfactant, a sharper curve was obtained for the MC-P sample, indicating a narrow particle size distribution between 80 and 90 nm. In the case of the MC-G, according to the mechanism described earlier, very fine particles were synthesized in the range from 30 to 45 nm, causing the curve to shift to the left [27,28]. The presence of oleic acid, on the other hand, has resulted in larger Nano-disk with an average diameter of 90 nm and a relatively wide range from 75 to 105 nm [34,44].

Ultimately, comparing the results acquired in this research with other studies, it was discovered that by employing different surfactants, completely different products were obtained at a relatively reasonable expense using the same method. Some of them are summarized in Table 3. Comparing the findings of this investigation with those of other studies reveals that using adequate surfactants greatly reduced the size of the synthesized particles in addition to decreasing agglomeration. Additionally, MOH was produced in utterly distinct morphologies at the same temperature and time. Meanwhile, in other studies, expensive raw materials, extended processing times, or extremely high temperatures were employed.

## 4. Conclusions

The basic purpose of this research was to establish novel MOH synthesis methods that are fast, inexpensive, and energy efficient, as well as to enable control of particle size and morphology. In order to achieve different morphologies, MOH was synthesized from two precursors of MgO and MgCl${}_{2}$ using a hydrothermal method at 180 °C for 18 h with the addition of four surfactants: CTAB, PEG500, Gelatin, and Oleic Acid. XRD and FESEM analysis, both confirmed that MgO is not an upright precursor at this temperature and time. However, using MgCl${}_{2}$ resulted in nanoparticles with hexagonal structure and plate, flake, spherical, and disc morphologies in the presence of different surfactants. This study showed that employing appropriate surfactants significantly reduced size, produced diverse morphologies, and decreased agglomeration. In contrast, other research used expensive raw materials, lengthy synthesis durations, or excessively high processing temperatures.

## Figures and Tables

**Table 1:** Surfactants used in the synthesis of MOH.

Surfactant	Chemical Name	Composition	Chemical Structure
CTAB	Cetrimonium bromide	$C_{19}H_{42}\mathrm{BrN}$	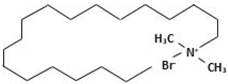
PEG500	Polyethylene Glycol	$C_{2}{}_{4}H_{5}{}_{0}O_{12}$	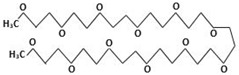
Gelatin	Collagens Polypeptide	$C_{102}H_{151}\;N_{31}O_{39}$	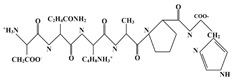
Oleic acid	(Z)-Octadec-9-enoic acid	$C_{18}H_{34}O_{2}$	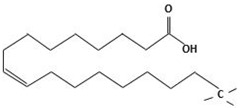

**Table 2:** Synthetic powders information.

Sample Code	Surfactant	Mg Precursor
MC	-	MgCl${}_{2}$
MC-C	CTAB	MgCl${}_{2}$
MC-P	PEG500	MgCl${}_{2}$
MC-G	Gelatin	MgCl${}_{2}$
MC-O	Oleic Acid	MgCl${}_{2}$
MO-C	CTAB	MgO
MO-P	PEG500	MgO
MO-G	Gelatin	MgO
MO-O	Oleic Acid	MgO

**Table 3:** Nanostructured Mg(OH)${}_{2}$ obtained under different hydrothermal conditions.

Mg Precursor	Solvent	Temperature (°C)	Duration (Hour)	Surfactant	Morphology	Particle Size (nm)	Ref.
MgCl${}_{2}$	H${}_{2}$O	180	18	CTAB PEG500 Gelatin Oleic Acid	Flake Plate Spherical Disk	79.86 75.09 40.07 90.54	This study
Mg	Ethanol (8:1) Ethanol (1:6)	180	20	-	Rod Lamellar	200 100	[13]
MgSO${}_{4}$	Ethanol (4:1) Ethanol (1:1)	180	20	-	Needle Lamellar	100 150	[13]
Mg(NO${}_{3}$)${}_{2}$	Ethylendiamine Ammonium hydroxide	180	20	-	Lamellar	100 50	[13]
Mg${}_{10}$(OH)${}_{18}$Cl${}_{2}$ (nano wire)	Ethylendiamine	180	6	-	Plate	160	[14]
Mg${}_{10}$(OH)${}_{18}$Cl${}_{2}$ (nano wire)	Ethanol (1:1)	180	6	-	Rod	300	[15]
MgCl${}_{2}$	NH${}_{3}$.H${}_{2}$O	250	36	-	Tube	100	[17]
MgCl${}_{2}$ in citric acid	NH${}_{3}$.H${}_{2}$O	120	5	MEA DEA TEA	Agglomerate	250	[18]
MgCl${}_{2}$	NH${}_{3}$.H${}_{2}$O	150	6	CTAB	Lamellar	400	[19]
MgO	H${}_{2}$O	200	24	CTAB	Rod aggregates	300	[20]
Mg(NO${}_{3}$)${}_{2}$	H${}_{2}$O	160	12	PEG-20000	Flake	100	[21]

## Data Availability

All data generated or analyzed during this study are included in this published article.

## References

[1] Patnaik P. (2003). Handbook of Inorganic Chemicals.

[2] Henrist C., Mathieu J.P., Vogels C., Rulmont A., Cloots R. (2003). Morphological study of magnesium hydroxide nanoparticles precipitated in dilute aqueous solution. J. Cryst. Growth.

[3] Zhao Z., Schlexer Lamoureux P., Kulkarni A., Bajdich M. (2019). Trends in Oxygen Electrocatalysis of 3 d-Layered (Oxy)(Hydro) Oxides. ChemCatChem.

[4] Feng S.H., Li G.H. (2017). Hydrothermal and solvothermal syntheses. Modern Inorganic Synthetic Chemistry.

[5] Balducci G. (2015). Lightweight Metal Hydride–Hydroxide Systems for Solid State Hydrogen Storage. Ph.D. Thesis.

[6] Hanlon J.M., Diaz L.B., Balducci G., Stobbs B.A., Bielewski M., Chung P., MacLaren I., Gregory D.H. (2015). Rapid surfactant-free synthesis of Mg(OH)_2_ nanoplates and pseudomorphic dehydration to MgO. CrystEngComm.

[7] Kumar Y.A., Kumar K.D., Kim H.J. (2020). Reagents assisted ZnCo_2_O_4_ nanomaterial for supercapacitor application. Electrochim. Acta.

[8] Kim H.J., Naresh B., Cho I.H., Bak J.S., Hira S.A., Reddy P.S., Krishna T., Kumar K.D., Mola B.A., Kumar Y.A. (2021). An advanced nano-sticks & flake-type architecture of manganese-cobalt oxide as an effective electrode material for supercapacitor applications. J. Energy Storage.

[9] Kumar Y.A., Sambasivam S., Hira S.A., Zeb K., Uddin W., Krishna T., Kumar K.D., Obaidat I.M., Kim H.J. (2020). Boosting the energy density of highly efficient flexible hybrid supercapacitors via selective integration of hierarchical nanostructured energy materials. Electrochim. Acta.

[10] Moniruzzaman M., Anil Kumar Y., Pallavolu M.R., Arbi H.M., Alzahmi S., Obaidat I.M. (2022). Two-dimensional core-shell structure of cobalt-doped@ MnO_2_ nanosheets grown on nickel foam as a binder-free battery-type electrode for supercapacitor application. Nanomaterials.

[11] Chinthala M., Balakrishnan A., Venkataraman P., Manaswini Gowtham V., Polagani R.K. (2021). Synthesis and applications of nano-MgO and composites for medicine, energy, and environmental remediation: A review. Environ. Chem. Lett..

[12] Yousefi S., Ghasemi B., Nikolova M.P. (2021). Opto-structural characterization of Mg(OH)_2_ and MgO nanostructures synthesized through a template-free sonochemical method. Appl. Phys. A.

[13] Ding Y., Zhang G., Wu H., Hai B., Wang L., Qian Y. (2001). Nanoscale magnesium hydroxide and magnesium oxide powders: Control over size, shape, and structure via hydrothermal synthesis. Chem. Mater..

[14] Fan W., Sun S., You L., Cao G., Song X., Zhang W., Yu H. (2003). Solvothermal synthesis of Mg(OH)_2_ nanotubes using Mg_10_(OH)_18_Cl_2_·5H_2_O nanowires as precursors. J. Mater. Chem..

[15] Fan W., Sun S., Song X., Zhang W., Yu H., Tan X., Cao G. (2004). Controlled synthesis of single-crystalline Mg(OH)_2_ nanotubes and nanorods via a solvothermal process. J. Solid State Chem..

[16] Jeevanandam P., Mulukutla R., Yang Z., Kwen H., Klabunde K. (2007). Nanocrystals to nanorods: A precursor approach for the synthesis of magnesium hydroxide nanorods from magnesium oxychloride nanorods starting from nanocrystalline magnesium oxide. Chem. Mater..

[17] Zhuo L., Ge J., Cao L., Tang B. (2009). Solvothermal synthesis of CoO, Co_3_O_4_, Ni(OH)_2_ and Mg(OH)_2_ nanotubes. Cryst. Growth Des..

[18] Chen Y., Zhou T., Fang H., Li S., Yao Y., He Y. (2015). A novel preparation of nano-sized hexagonal Mg(OH)_2_. Procedia Eng..

[19] Yan H., Zhang X.H., Wu J.M., Wei L.Q., Liu X.G., Xu B.S. (2008). The use of CTAB to improve the crystallinity and dispersibility of ultrafine magnesium hydroxide by hydrothermal route. Powder Technol..

[20] Dhaouadi H., Chaabane H., Touati F. (2011). Mg(OH)_2_ nanorods synthesized by a facile hydrothermal method in the presence of CTAB. Nano-Micro Lett..

[21] Wang Q., Li C., Guo M., Sun L., Hu C. (2014). Hydrothermal synthesis of hexagonal magnesium hydroxide nanoflakes. Mater. Res. Bull..

[22] Zhu Y.J., Chen F. (2014). Microwave-assisted preparation of inorganic nanostructures in liquid phase. Chem. Rev..

[23] Naidu B.N., Kumar K.L., Saini H., Kumar M., Kumar T.N., Prasad V. (2022). Coke deposition over Ni-based catalysts for dry reforming of methane: Effects of MgO-Al_2_O_3_ support and ceria, lanthana promoters. J. Environ. Chem. Eng..

[24] Liu S., Wei X., Lin S., Guo M. (2021). Preparation of aerogel Mg(OH)_2_ nanosheets by a combined sol–gel-hydrothermal process and its calcined MgO towards enhanced degradation of paraoxon pollutants. J. Sol-Gel Sci. Technol..

[25] Käselau S., Scheel S., Petersson L., Ho C.H., Luinstra G.A. (2018). Synthesis of a linear low-density polyethylene/MgO@ Mg(OH)_2_ nanocomposite using modified in situ polymerization. Polym. Int..

[26] Yun L., Wang B., Jing D., Lv X., Yu C., Wang G., Huang L., Mujumdar A.S. (2009). Drying kinetics of magnesium hydroxide of different morphological micro nanostructures. Dry. Technol..

[27] Mochane M.J., Mokhothu T.H., Mokhena T.C. (2022). Synthesis, mechanical, and flammability properties of metal hydroxide reinforced polymer composites: A review. Polym. Eng. Sci..

[28] Sharma D., Ledwani L., Kumar N., Pervaiz N., Mehrotra T., Kumar R. (2020). Structural and physicochemical properties of Rheum emodi mediated Mg (OH)_2_ nanoparticles and their antibacterial and cytotoxic potential. IET Nanobiotechnol..

[29] Meng W., Wu H., Wu R., Wang T., Wang A., Ma J., Xu J., Qu H. (2021). Fabrication of surface-modified magnesium hydroxide using Ni^2+^ chelation method and layer-by-layer assembly strategy: Improving the flame retardancy and smoke suppression properties of ethylene-vinyl acetate. Colloids Surf. A Physicochem. Eng. Asp..

[30] Yao D., Yin G., Bi Q., Yin X., Wang N., Wang D.Y. (2020). Basalt fiber modified ethylene vinyl acetate/magnesium hydroxide composites with balanced flame retardancy and improved mechanical properties. Polymers.

[31] Zheng T., Xia W., Guo J., Liu Y. (2020). Modified magnesium hydroxide encapsulated by melamine cyanurate in flame-retardant polyamide-6. J. Polym. Res..

[32] Wu H., Luo B., Gao C., Wang L., Wang Y., Zhang Q. (2020). Synthesis and size control of monodisperse magnesium hydroxide nanoparticles by microemulsion method. J. Dispers. Sci. Technol..

[33] Bukhtiyarova M. (2019). A review on effect of synthesis conditions on the formation of layered double hydroxides. J. Solid State Chem..

[34] Chen Y., Zhou T., Fang H., Li S., Yao Y., Fan B., Wang J. (2016). A novel preparation of nanosized hexagonal Mg(OH)_2_ as a flame retardant. Particuology.

[35] Byrappa K., Adschiri T. (2007). Hydrothermal technology for nanotechnology. Prog. Cryst. Growth Charact. Mater..

[36] Li Y., Sui M., Ding Y., Zhang G., Zhuang J., Wang C. (2000). Preparation of Mg(OH)_2_ nanorods. Adv. Mater..

[37] Ruhaimi A.H., Ab Aziz M.A. (2021). High-performance flake-like mesoporous magnesium oxide prepared by eggshell membrane template for carbon dioxide capture. J. Solid State Chem..

[38] Yedluri A.K., Kim H.J. (2018). Wearable super-high specific performance supercapacitors using a honeycomb with folded silk-like composite of NiCo_2_O_4_ nanoplates decorated with NiMoO_4_ honeycombs on nickel foam. Dalton Trans..

[39] Arbi H.M., Yadav A.A., Anil Kumar Y., Moniruzzaman M., Alzahmi S., Obaidat I.M. (2022). Polypyrrole-Assisted Ag Doping Strategy to Boost Co(OH)_2_ Nanosheets on Ni Foam as a Novel Electrode for High-Performance Hybrid Supercapacitors. Nanomaterials.

[40] Kumar Y.A., Das H.T., Guddeti P.R., Nallapureddy R.R., Pallavolu M.R., Alzahmi S., Obaidat I.M. (2022). Self-Supported Co_3_O_4_@ Mo-Co_3_O_4_ Needle-like Nanosheet Heterostructured Architectures of Battery-Type Electrodes for High-Performance Asymmetric Supercapacitors. Nanomaterials.

[41] Yousefi S., Ghasemi B. (2019). Ultrasound-assisted synthesis of porous Mg(OH)_2_ nanostructures using hypersaline brine. Micro Nano Lett..

[42] Jeevanandam J., San Chan Y., Danquah M.K. (2020). Effect of pH variations on morphological transformation of biosynthesized MgO nanoparticles. Part. Sci. Technol..

[43] Yousefi S., Ghasemi B. (2021). Mg(OH)_2_ nanostructures using impure brine: Optimization of synthesis parameters by Taguchi robust design and study of optical properties. Res. Chem. Intermed..

[44] Sohrabi-Kashani L., Yekta B.E., Rezaie H.R., Zolriasatein A. (2022). Synergistic effect of micro-and nano-TiO_2_ on hydrophobic, mechanical, and electrical properties of hybrid polyurethane composites. J. Mater. Sci. Mater. Electron..

